# Endoscopic ultrasound-guided sigmoid enterocolostomy for malignant bowel obstruction in frozen pelvis

**DOI:** 10.1055/a-2883-0502

**Published:** 2026-06-16

**Authors:** Zishuang Zhou, Xujie Zhou, Lilan Fan, Hongling Wang

**Affiliations:** 1 Department of Gastroenterology 89674 Zhongnan Hospital of Wuhan UniversityWuhanHubeiChina; 2 Hubei Clinical Center and Key Lab of Intestinal and Colorectal DiseasesWuhanChina

**Keywords:** Endoscopy Lower GI Tract, Stenting, Endoscopic ultrasonography, Intervention EUS

**Fig. 1:**
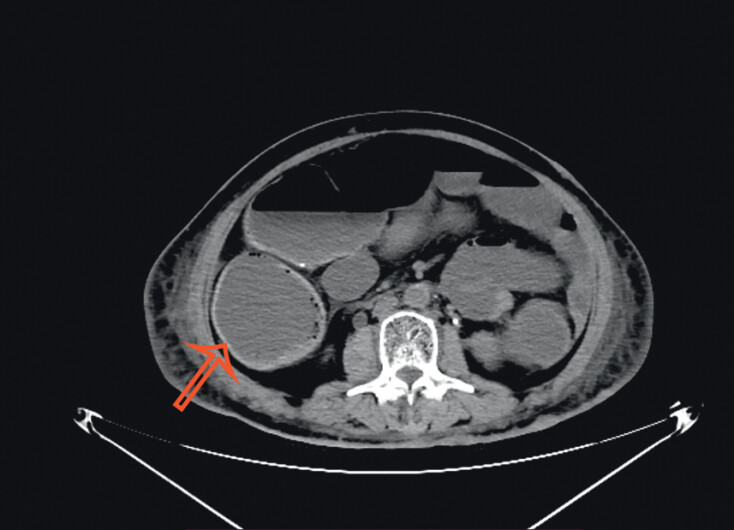
Preoperative abdominal CT showing markedly dilated bowel loops secondary to a frozen pelvis. The red arrow indicates the dilated target bowel loop.

A 52-year-old woman with recurrent cervical cancer after surgery and radiotherapy presented with nausea, vomiting, acute kidney injury, and obstructive nephropathy. Computed tomography (CT) showed marked small-bowel and colonic dilatation, multiple pelvic metastases, and a frozen pelvis with fixed angulated bowel loops ( Fig. 1 ). Surgical diversion was considered high risk after a multidisciplinary review, and conventional transanal self-expandable metal stent placement was unlikely to be feasible because the stable guidewire support across the distorted segment could not be achieved. 1​ 2​ ​3​

**Fig. 2:**
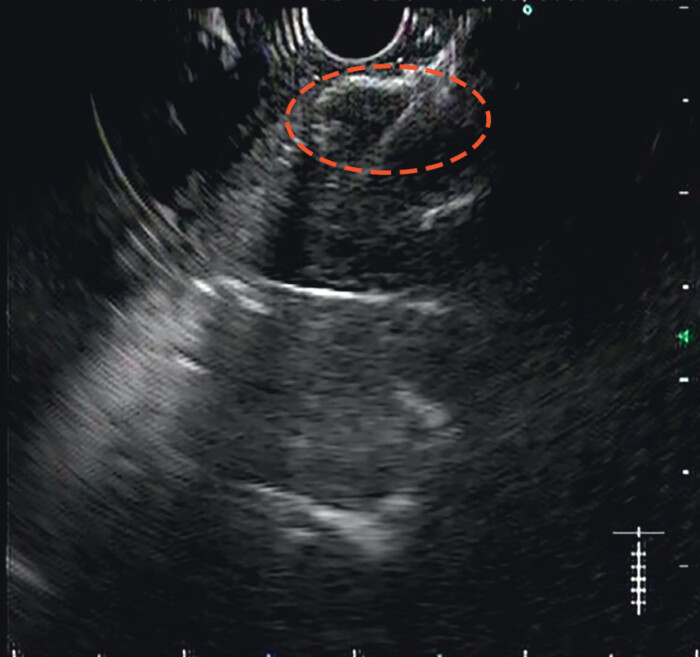
EUS-guided puncture of the dilated proximal bowel loop from the sigmoid colon. The red dashed circle indicates the targeted dilated bowel loop.

**Fig. 3:**
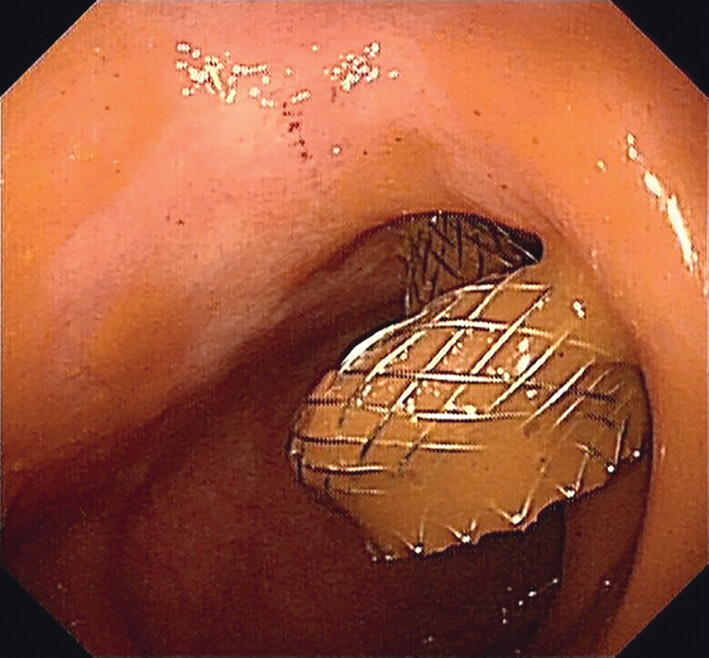
A direct endoscopic view after lumen-apposing metal stent deployment, showing the proximal flange fully deployed in the sigmoid colon.

After decompression with a transnasal ileus tube, EUS-guided enterocolostomy (EUS-EC) was performed from the sigmoid colon. Preprocedural imaging showed close apposition between the sigmoid colon and the dilated proximal bowel loop, creating a suitable puncture window ( Fig. 2 ). Under EUS guidance, the target bowel was punctured with a 22-gauge needle, contrast confirmed intraluminal access, and a 15 mm×10 mm lumen-apposing metal stent was deployed ( Fig. 3 ), to create an internal bypass. Immediate drainage of intestinal contents was observed ( Video 1 ).

**Fig. 4:**
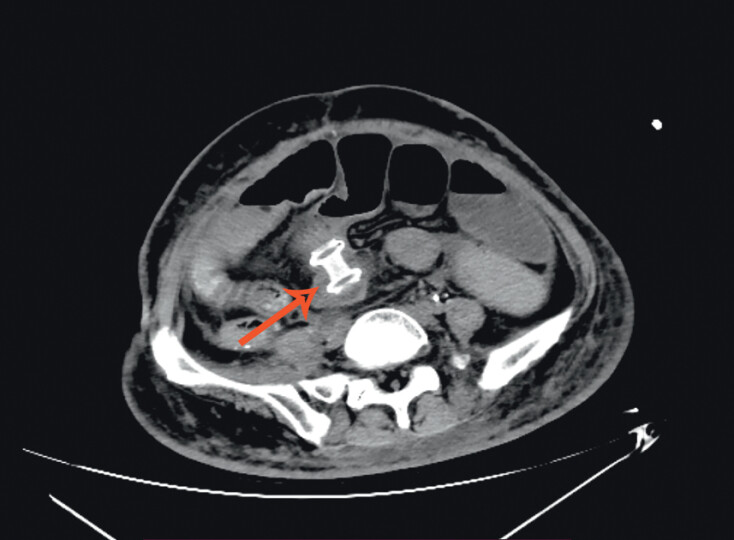
EUS-guided sigmoid enterocolostomy with lumen-apposing metal stent deployment for MBO in a frozen pelvis, showing targeted puncture, stent release, and immediate decompression.

The procedure lasted 90 minutes. Abdominal distension improved within 24 hours, with an approximately 4-cm reduction in abdominal circumference. No intraprocedural bleeding, perforation, or stent migration occurred. Follow-up CT on postoperative day 2 confirmed the stent in situ and showed reduced bowel dilatation and air-fluid levels ( Fig. 4 ). The patient was transferred for palliative supportive care on postoperative day 6 and died 30 days later from tumor progression. In selected patients with a frozen pelvis and malignant bowel obstruction, EUS-EC may provide an effective minimally invasive palliation option when surgery or transanal stenting is not suitable. 4​ ​5​

**Video 1** Postoperative abdominal CT confirming the correct stent position and reduced obstructive burden. The red arrow indicates the lumen-apposing metal stent in situ.


Endoscopy_UCTN_Code_TTT_1AS_2AK

Informed Consent

Written informed consent was obtained from the patient to publish these images and the video.
